# Supplementary material for: Selective oxidative protection leads to tissue topological changes orchestrated by macrophage during ulcerative colitis
Source: Nat Commun. 2023 Jun 21;14:3675. doi: 10.1038/s41467-023-39173-2 (PMC10284839; doi:10.1038/s41467-023-39173-2)
Supplement: Supplementary file 1 — Supplementary Information [file 41467_2023_39173_MOESM1_ESM.pdf]

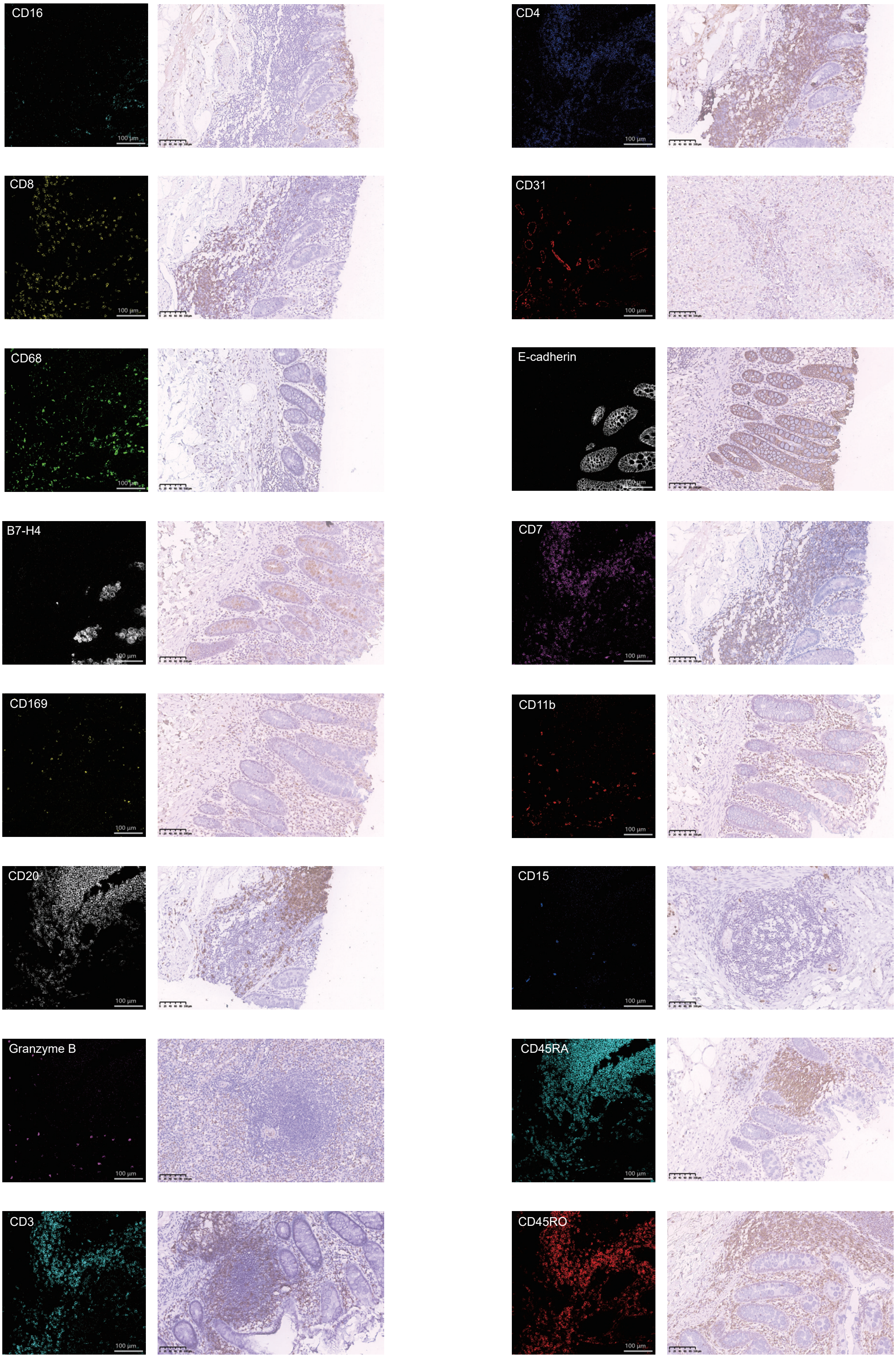

Figure S1-3. Screening and validation of IMC Antibodies, related to Figure 1
Antibodies for conjugation with metals were tested individually by standard IHC using the same, non-conjugated antibody clone on UC sections. Clones, sources, and detailed information were listed for each antibody in the sheet of Imaging Mass Cytometry Antibody panel in Table S1. Examples of IMC staining (the left single-color images) and similar areas of independent IHC staining images were shown (the right colorful images). Brightness and contrast adjusted. Scale bars, 100 µm. Representative plot from 52 samples (2 independent experiments).
Figure S1

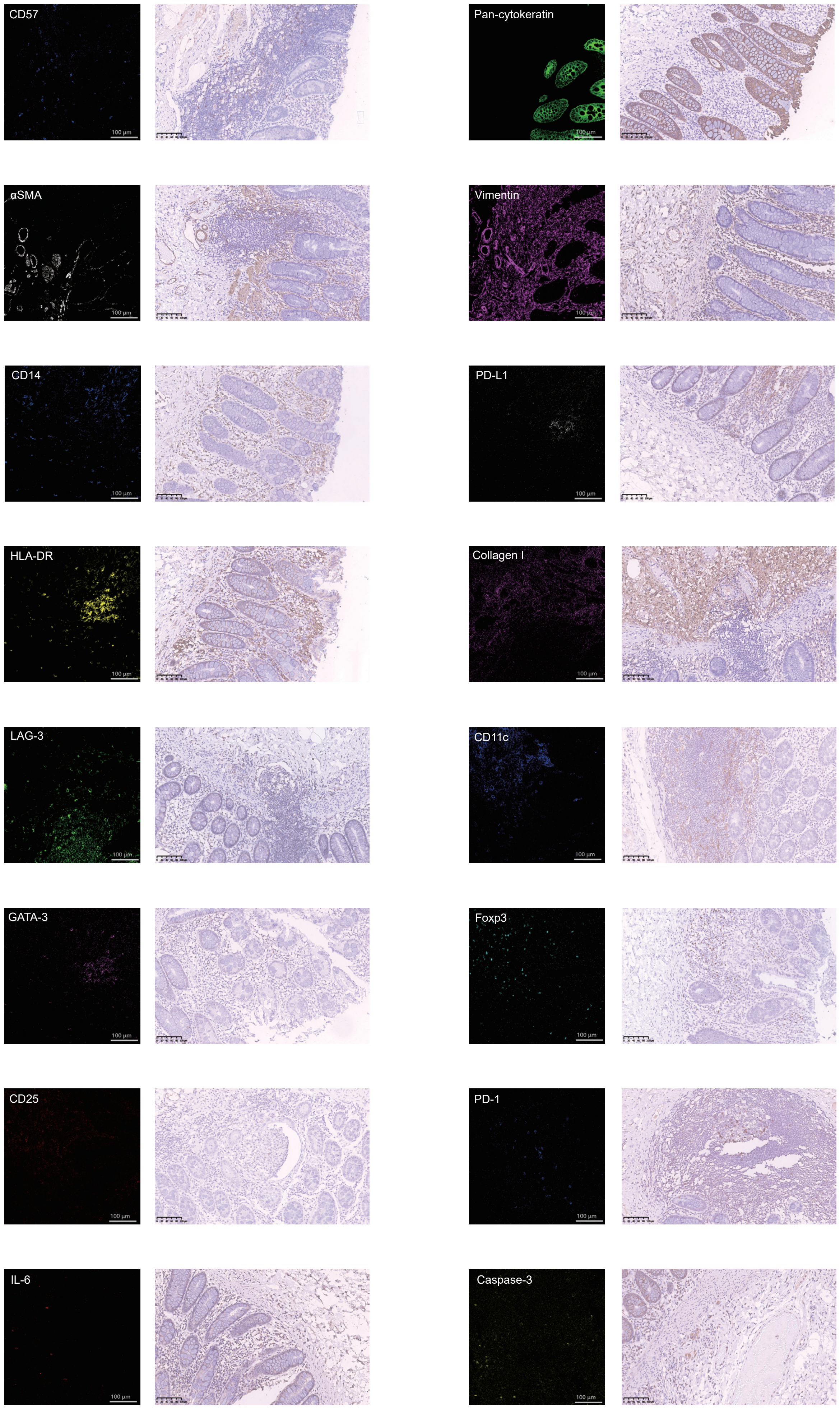
CD57
Pan-cytokeratin
αSMA
Vimentin
CD14
PD-L1
HLA-DR
Collagen I
LAG-3
CD11c
GATA-3
Foxp3
CD25
PD-1
IL-6
Caspase-3

Figure S2

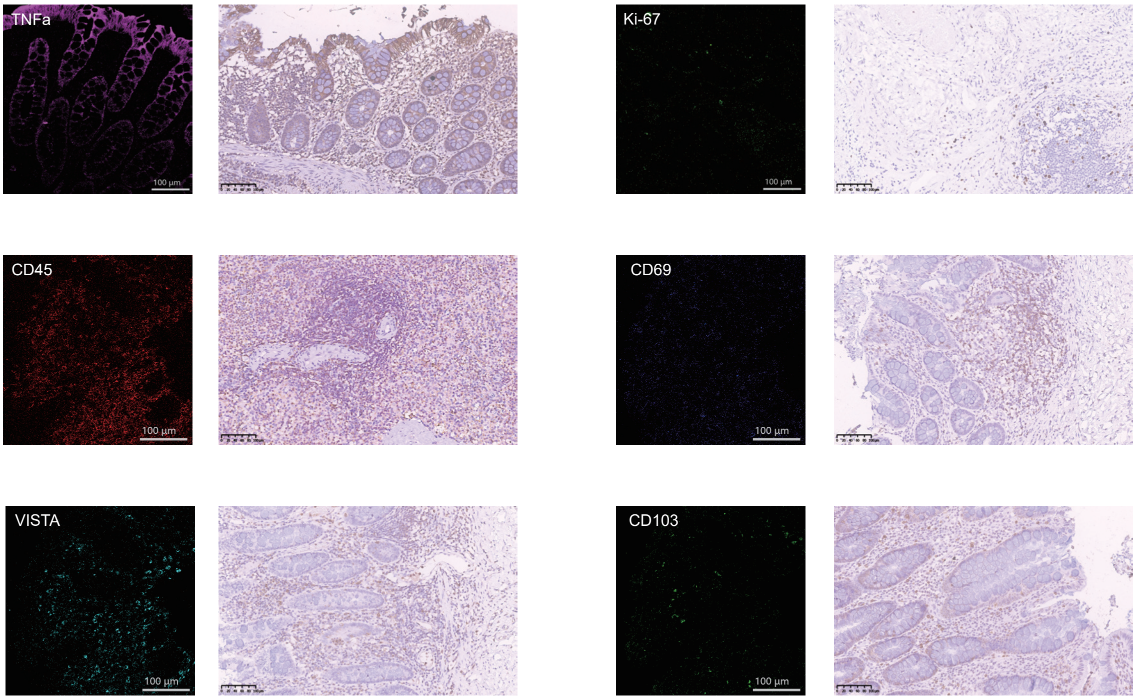
TNFa
100 µm
Ki-67
100 µm
CD45
100 µm
CD69
100 µm
VISTA
100 µm
CD103
100 µm

Figure S3

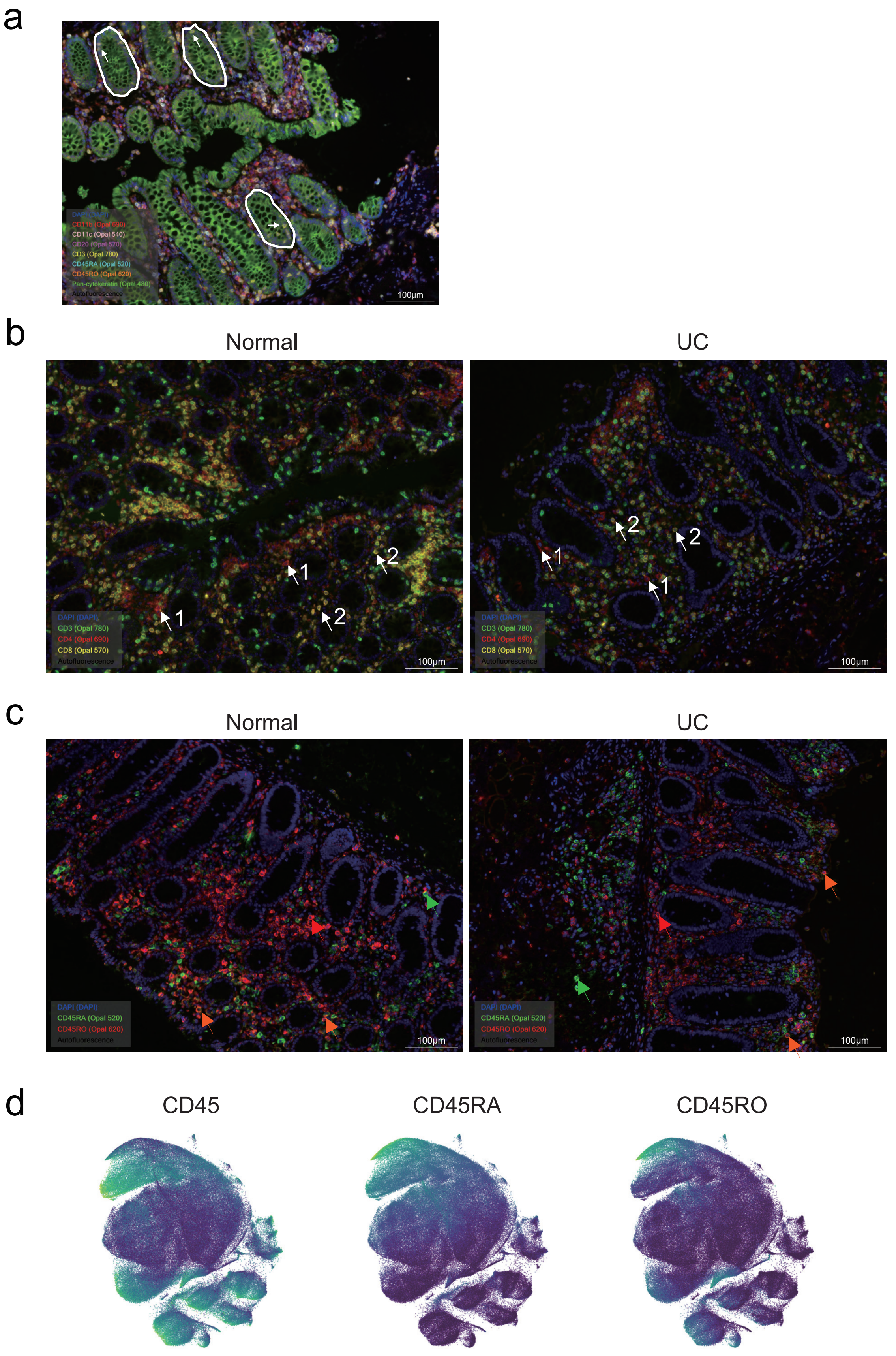

Figure S4 Validation of IMC staining via mIHC.
a. Intestinal glands marked by white circles are highlighted with Pan-cytokeratin (green). White arrows point out T cells stained with CD3 (yellow) which are located at the glands. All markers are listed in the legend on the image. Scale bars, 100 µm. Representative plot from 5 samples (2 independent experiments).
b. Arrows 1 point out CD4 (red) cells and arrows 2 label CD8 (yellow) cells which are all stained without CD3 (green) antibodies in UC and normal sections. Scale bars, 100 µm. Representative plot from 5 samples (2 independent experiments).
c. The arrows mark the cells simultaneously expressing CD45RA (green) and CD45RO (red). Scale bars, 100 µm. Representative plot from 5 samples (2 independent experiments).
d. The Cytof data of the UC sample shows the expression of CD45, CD45RA, and CD45RO which represent the co-staining of CD45RA and CD45RO.

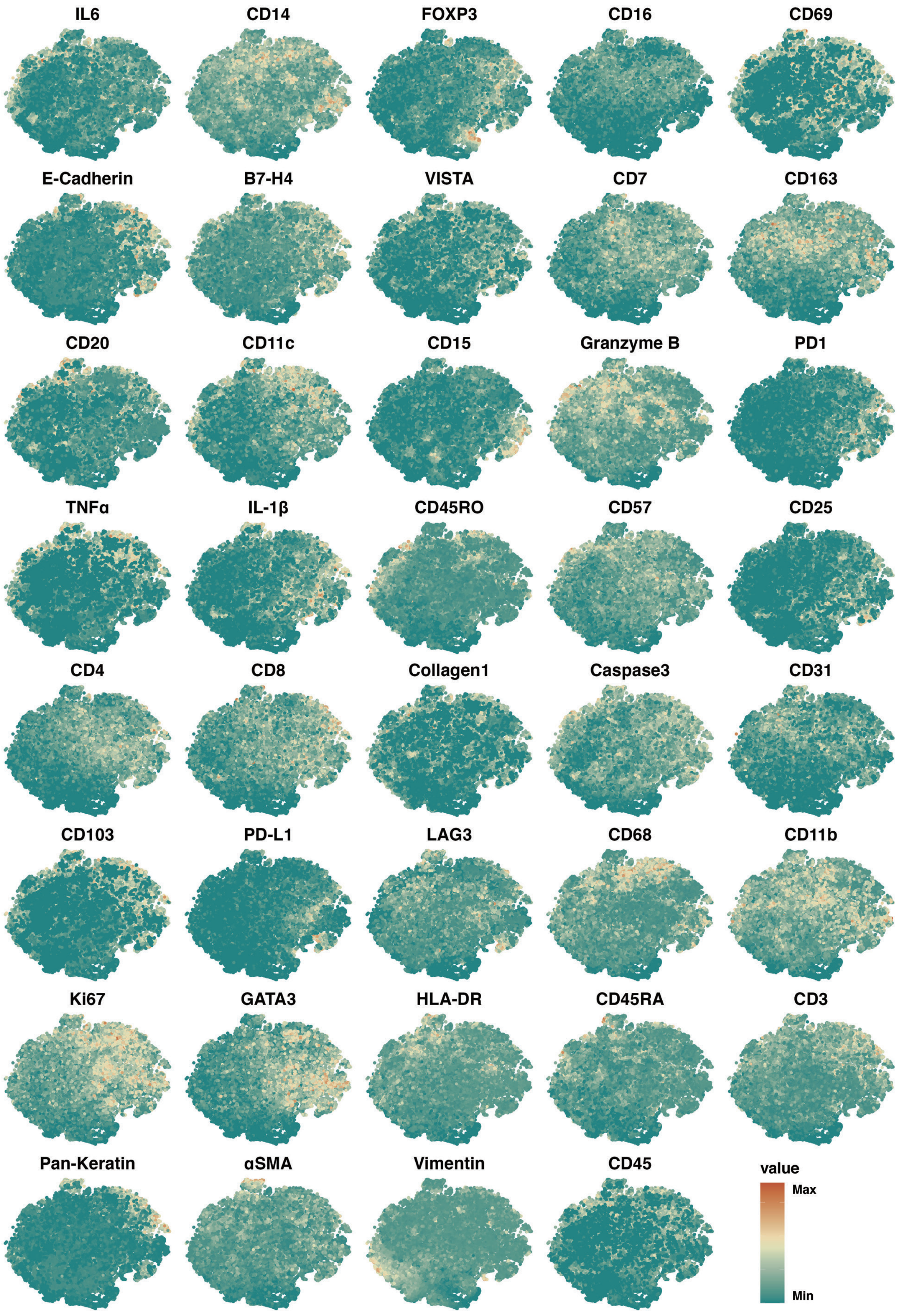

Figure S5. tSNE plot showing expression patterns of all 39 protein markers in IMC analysis

a

ID	Key markers
Vimentin$^+$ Mesenchymal Cells	Vimentin
Lineage$^-$ Cells	no obvious lineage marker
Tregs	Foxp3
Resident Macrophages	CD11b^{low} CD163$^+$
Epithelials/Immune Suppressive Cells	Epi (E-cad$^+$CD103$^+$), immunosuppressive: Treg (CD4$^+$FoxP3$^+$CD25$^+$), immunuosuppresive markers(PD-L1$^+$VISTA$^+$B7-H4$^+$)
Gata3$^+$ T Cells	Gata3$^+$CD4$^+$CD8$^+$
Gata3+ CD8+ TCells	Gata3$^+$CD8$^+$
Exhaustic T Cells	CD4$^+$CD8$^+$PD-1$^+$VISTA$^+$
Neutrophils	CD15
Infiltrating Macrophages/TNFα+ T Cells	InfM(CD11b$^+$CD68$^+$CD14$^+$)+TNFα$^+$ T (CD45RO$^+$CD4$^+$CD8$^+$TNFα$^+$)
TNFα+ B and NK	TNFa$^+$ B (CD20$^+$) and NK (CD57$^+$)
Infiltrating Macrophages/TNFα+ T Cells/NK	InfM (CD11b$^+$CD68$^+$) and TNFα$^+$T (CD4$^+$CD8$^+$) and NK (CD57$^+$)
Infiltrating Macrophages	CD11b$^+$CD68$^+$CD163$^+$CD14$^+$
TNFα+ Epithelials	TNFa$^+$PanCK$^+$
Fibroblasts/Endothelials	CD31$^+$ and αSMA$^+$
Resident Macrophages/Epithelials	CD11^{blow}CD163$^+$CD14$^+$ and E-cad$^+$CD103$^+$
Epithelials	Pan-CK

b

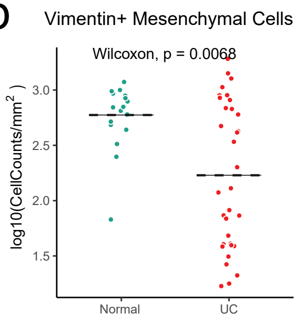

c

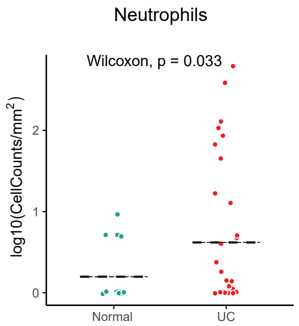

Figure S6 Cell cluster annotation

a. Key markers of each CT for IMC cluster annotation.

b-c. Dot plots showing the abundance of b) Vimentin+ mesenchymal cells and c) neutrophils between normal and UC samples（Normal, n=19; UC, n=33 from two independent experiments）. Dashed black line labeling mean value of each group and statistical significance were performed with two-sided Wilcoxon rank-sum tests. Exact P values were provided in the plot and Source Data file.

Imaging Mass Cytometry Antibody Panel							
Antibody	Metal	Clone	Source (antibody)	Cat# (antibody)	Source (metal)	Cat# (metal)	Dilution (Stock solution 0.5 mg/ml)
CD45	89 Y	D9M8I	CST	13917S	Macklin	Y820645	1:50
IL6	115 In	1A3B4	Proteintech	66146-1-Ig	Macklin	I822392	1:50
CD14	141 Pr	EPR3653	Abcam	ab226121	Fluidigm	201141A	1:100
FoxP3	142 Pr	D6O8R	CST	12653S	Fluidigm	201142A	1:50
CD16	143 Nd	EPR16784	Abcam	ab256582	Fluidigm	201143A	1:100
CD69	144 Nd	EPR21814	Abcam	ab234512	Fluidigm	201144A	1:50
CD4	145Nd	EPR6855	Abcam	ab181724	Fluidigm	201145A	1:100
CD8a	146 Nd	C8/144B	Biolegend	372902	Fluidigm	201146A	1:100
Collagen I	147 Sm	EPR7785	Abcam	ab215969	Fluidigm	201147A	1:100
Cleaved Caspase-3	148 Nd	5A1E	CST	9664S	Fluidigm	201148A	1:50
CD31	149 Sm	89C2	CST	3528S	Fluidigm	201149A	1:100
E-cadherin	150 Nd	DECMA-1	Biolegend	147302	Fluidigm	201150A	1:100
B7-H4	151 Eu	H74	Thermofisher	14-5949-82	Fluidigm	201151A	1:50
VISTA	152 Sm	D1L2G	CST	64953S	Fluidigm	201152A	1:50
CD7	153 Eu	EPR4242	Abcam	ab230834	Fluidigm	201153A	1:100
CD169	154 Sm	HSn 7D2	Abcam	ab18619	Fluidigm	201154A	1:100
CD103	155 Gd	EPR4166(2)	Abcam	ab271889	Fluidigm	201155A	1:100
PD-L1	156 Gd	E1L3N	CST	13684S	Fluidigm	201156A	1:50
LAG3	158 Gd	D2G4O	CST	15372S	Fluidigm	201158A	1:50
CD68	159 Tb	C8/144B	Biolegend	372902	Fluidigm	201159A	1:400
CD11b	160 Gd	EPR1344	Abcam	ab209970	Fluidigm	201160A	1:200
CD20	161 Dy	IGEL/773	Abcam	ab213033	Fluidigm	201161A	1:100
CD11c	162 Dy	EP1347Y	Abcam	ab216655	Fluidigm	201162A	1:100
CD15	163 Dy	HI98	BD	563872	Fluidigm	201163A	1:100
Granzyme B	164 Dy	EPR20129-217	Abcam	ab219803	Fluidigm	201164A	1:100
PD-1	165 Ho	D4W2J	CST	86163S	Fluidigm	201165A	1:50
Ki67	166 Er	B56	BD	550609	Fluidigm	201166A	1:100
GATA-3	167 Er	D13C9	CST	5852S	Fluidigm	201167A	1:50
HLA-DR	168 Er	EDHu-1	Novus	NB110-40686	Fluidigm	201168A	1:200
CD45RA	169 Tm	HI100	Biolegend	304143	Fluidigm	201169A	1:200
CD3ε	170 Er	D7A6E	CST	85061S	Fluidigm	201170A	1:100
TNFα	171 Yb	7B8A11	Proteintech	60291-1-Ig	Fluidigm	201171A	1:50
IL-1β	172 Yb	2A1B4	Proteintech	66737-1-Ig	Fluidigm	201172A	1:50
CD45RO	173 Yb	UCHL1	Biolegend	304239	Fluidigm	201173A	1:200
CD57	174 Yb	NK-1	BD	555618	Fluidigm	201174A	1:100
IL-2 Receptor alpha (CD25)	175 Lu	EPR6452	Abcam	ab215378	Fluidigm	201175A	1:50
Pan-Cytokeratin	176 Yb	AE-1/AE-3	Biolegend	914204	Fluidigm	201176A	1:200
Alpha Smooth Muscle Actin	194 Pt	1A4	Biolegend	904601	Fluidigm	201194	1:100
Vimentin	198 Pt	D21H3	CST	5741S	Fluidigm	201198	1:200
**Table S1**							

mIHC Antibody Panel

Species	Antibodies	Clone	Source	Cat#	Dilution
Human	Pan-cytokeratin	AE-1/AE-3	biolegend	914204	1:100
Human	CD3e	D7A6E	CST	85061S	1:100
Human	CD11b	EPR1344	abcam	ab209970	1:200
Human	CD20	H1	BD	555677	1:100
Human	CD11c	EP1347Y	abcam	ab216655	1:100
Human	CD45RO	UCHL1	biolegend	304239	1:200
Human	CD45RA	HI100	biolegend	304143	1:200
Human	CD4	EPR6855	abcam	ab181724	1:100
Human	CD8	C8/144B	biolegend	372902	1:100
Human	CD45	D9M8I	CST	13917BF	1:100
Human	TNF-α	7B8A11	Proteintech	60291-1-Ig	1:100

Table S1

Human CyTOF Antibody Panel							
**Antibodies**	**Metal**	**Clone**	**Source (antik**	**Cat# (antibody)**	**Source (metal)**	**Cat# (metal)**	**Dilution**
CD45	89Y	HI30	Biolegend	304045	Macklin	Y820645	1:100
CD3	115In	UCHT1	Biolegend	300443	Macklin	I811750	1:100
Ki-67	139La	SolA15	Biolegend	350523	Macklin	L812522	1:100
CD56	141Pr	HCD56	Biolegend	318345	Fluidigm	201141A	1:100
TCR gd	142Nd	5A6.E9	Thermofisher	TCR1061	Fluidigm	201142A	1:100
CCR6	143Gd	G034E3	Biolegend	353427	Fluidigm	201143A	1:100
CD134	144Nd	BER-ACT35	Biolegend	350015	Fluidigm	201144A	1:100
IgD	145Nd	IA6-2	Biolegend	348235	Fluidigm	201145A	1:100
CD7	146Nd	CD7-6B7	Biolegend	343111	Fluidigm	201146A	1:100
Tim-3	147Sm	F38-2E2	Biolegend	345019	Fluidigm	201147A	1:100
CD19	148Nd	HIB19	Biolegend	302247	Fluidigm	201148A	1:100
CD25	149Sm	M-A251	Biolegend	356102	Fluidigm	201149A	1:100
CD223	150Nd	874501	R&D	MAB23193	Fluidigm	201150A	1:100
CD20	151Eu	2H7	Biolegend	302343	Fluidigm	201151A	1:100
CD103	152Sm	B-Ly7	Thermofisher	14-1038-82	Fluidigm	201152A	1:100
CD161	153Eu	HP-3G10	Biolegend	339919	Fluidigm	201153A	1:100
TIGIT	154Sm	A15153G	Biolegend	372702	Fluidigm	201154A	1:100
CD45RA	155Gd	HI100	Biolegend	304143	Fluidigm	201155A	1:100
CCR4	156Gd	L291H4	Biolegend	359402	Fluidigm	201156A	1:100
CD27 (metal-labled)	157Nd	O323	PLTTECH	02.01.H.0000037			1:100
CCR7	158Gd	G043H7	Biolegend	353237	Fluidigm	201158A	1:100
GITR	159Tb	621	Biolegend	311602	Fluidigm	201159A	1:100
CD28	160Gd	CD28.2	Biolegend	302937	Fluidigm	201160A	1:100
CTLA4	161Dy	14D3	Thermofisher	14-1529-82	Fluidigm	201161A	1:100
Foxp3	162Dy	PCH101	Thermofisher	14-4776-82	Fluidigm	201162A	1:100
CD57	164Dy	HNK-1	Biolegend	359602	Fluidigm	201164A	1:100
Tbet	165Ho	4B10	Biolegend	644825	Fluidigm	201165A	1:100
CXCR3	166Er	G025H7	Biolegend	353733	Fluidigm	201166A	1:100
ICOS	167Er	C398.4A	Biolegend	313502	Fluidigm	201167A	1:100
4-1BB	168Er	4B4-1	Biolegend	309802	Fluidigm	201168A	1:100
CD45RO	169Tm	UCHL1	Biolegend	304239	Fluidigm	201169A	1:100
CD127	170Er	A019D5	Biolegend	351337	Fluidigm	201170A	1:100
CD69	171Yb	FN50	Biolegend	310939	Fluidigm	201171A	1:100
CD138	172Yb	DL-101	Biolegend	352311	Fluidigm	201172A	1:100
GranzymeB	173Yb	GB11	Thermofisher	MA1-80734	Fluidigm	201173A	1:100
PD-1	174Yb	EH12.2H7	Biolegend	329941	Fluidigm	201174A	1:100
BTLA	175Lu	MIH26	Biolegend	344502	Fluidigm	201175A	1:100
HLA-DR	176Yb	L243	Biolegend	307651	Fluidigm	201176A	1:200
CD4 (metal-labled)	197Au	RPA-T4	PLTTECH	02.01.H.0000643			1:100
CD8	198Pt	RPA-T8	Biolegend	301053	Fluidigm	201198	1:100

Table S1

FACS Antibody Panel					
**Species**	**Antibodies**	**Clone**	**Source**	**Cat#**	**Dilution**
human	CD45	HI30	BD	563716	1:600
human	CD11b	1CRF44	BD	562721	1:600
human	CD4	RPA-T4	BD	557871	1:600
human	CD8	RPA-T8	BD	557746	1:600
human	CD19	SJ25C1	BD	563549	1:600
human	CD3	SK7	Biolegend	981002	1:600
human	CD68	Y1/82A	Biolegend	333808	1:500
human	HLA-DR	L243	Biolegend	307626	1:600
human	EpCAM	9C4	Biolegend	324208	1:600
human	CCR2	K036C2	Biolegend	357204	1:600
mouse	CD45	30-F11	BD	563053	1:600
mouse	CD11b	M1/70	BD	562287	1:600
mouse	MHCII (I-A/I-E)	M5/114.15.2	BD	562564	1:500
mouse	CD4	GK1.5	BD	552051	1:600
mouse	F4/80	BM8	Biolegend	123114	1:600
mouse	CD3ε	17A2	Biolegend	100203	1:600
mouse	Ly6C	HK1.4	Biolegend	128036	1:600
mouse	Ly6G	1A8	Biolegend	127605	1:600
mouse	CD8a	53-6.7	Biolegend	100722	1:600
mouse	TNFα	MP6-XT22	Thermofisher	12-7321-82	1:100
**Table S1**					

Patient ID	Gender	Age	Diagnosis	Mayo Clinic Endoscopic Subscore	The Montreal classification	
					Extent (E1E2E3)	Severity
1	F	27	UC	1	1	mild
2	F	38	UC	2	2	moderate
3	M	43	UC	1	2	moderate
4	F	30	UC	1	2	mild
5	M	35	UC	2	3	moderate
6	M	57	UC	2	3	severe
7	M	53	UC	2	2	severe
8	M	45	UC	0	1	clinical remission
9	M	62	UC	3	2	mild
10	M	63	UC	1	2	moderate
11	F	66	UC	2	2	mild
12	F	37	UC	2	2	moderate
13	M	63	UC	3	2	severe
14	M	45	UC	3	3	moderate
15	M	45	UC	2	1	moderate
16	F	37	UC	3	2	severe
17	F	49	UC	3	1	mild
18	M	55	UC	2	3	clinical remission
19	F	60	UC	2	1	mild
20	F	65	UC	3	2	severe
21	M	38	UC	1	3	mild
22	M	48	UC	1	2	clinical remission
23	M	51	UC	2	1	moderate
24	F	70	UC	3	2	severe
25	M	66	UC	3	3	severe
26	M	70	UC	3	2	moderate
27	F	39	UC	3	2	severe
28	F	47	UC	1	1	severe
29	F	30	UC	2	2	mild
30	M	39	UC	2	1	moderate
31	M	27	UC	1	3	clinical remission
32	F	67	UC	2	2	mild
33	F	46	UC	3	3	severe
1	F	60	normal	0		
2	M	27	normal	0		
3	F	31	normal	0		
4	F	52	normal	0		
5	M	45	normal	0		
6	M	38	normal	0		
7	M	67	normal	0		
8	M	54	normal	0		
9	F	23	normal	0		
10	M	49	normal	0		
11	F	32	normal	0		
12	F	62	normal	0		
13	M	55	normal	0		
14	M	48	normal	0		

15	F	65	normal	0		
16	M	25	normal	0		
17	F	66	normal	0		
18	M	46	normal	0		
19	F	64	normal	0		
**Table S2**						

A Characteristics of UC patients and healthy controls for scRNAseq

Characteristics	Patient1	Patient 2	Patient 3	Patient 4	Patient 5	Control 1	Control 2	Control 3	Control 4
Age(years)	44	43	58	55	68	43	58	55	68
Gender(M/F)	M	F	M	M	M	F	M	M	M
Disease duration (years)	3	11	3.5	18	5				
Disease activity (adapted from Truelove& Witts).	Moderate	Moderate	Moderate	Moderate	Moderate				
Bloody stools/day	6	5	7	5	7				
Pulse(bpm)	76	87	78	72	88				
Temperature(℃)	36.6	37.1	36.8	36.6	36.5				
Hemoglobin(g/L)	103	106	155	151	132				
ESR(mm/h)	14	5	2	12	24				
CRP(mg/L)	5.8	10.2	3.9	1.9	16				
Mayo score									
Stool frequency	6	5	7	5	7				
Rectal bleeding	Obvious	Obvious	Obvious	Obvious	Obvious				
Mucosa	Moderate friab	Moderate friab	Moderate friab	Moderate friab	Moderate friability				
Physician’s global assessment	Moderate	Moderate	Moderate	Moderate	Moderate				

We deleted one UC sample (patient 2) and one self control (patient 1) sample because of lower percentage of mitochondrial genes and the lower estimated number of cells in the alignment.

Table S2
