# Supplementary material for: Selective oxidative protection leads to tissue topological changes orchestrated by macrophage during ulcerative colitis
Source: Nat Commun. 2023 Jun 21;14:3675. doi: 10.1038/s41467-023-39173-2 (PMC10284839; doi:10.1038/s41467-023-39173-2)
Supplement: Supplementary file 3 — Reporting Summary [file 41467_2023_39173_MOESM3_ESM.pdf]

nature portfolio

Corresponding author(s): Jianpeng Sheng

Last updated by author(s): Apr 3, 2023

Reporting Summary

Nature Portfolio wishes to improve the reproducibility of the work that we publish. This form provides structure for consistency and transparency in reporting. For further information on Nature Portfolio policies, see our Editorial Policies and the Editorial Policy Checklist.

Statistics

For all statistical analyses, confirm that the following items are present in the figure legend, table legend, main text, or Methods section.

n/a	Confirmed	
☐	☒	The exact sample size (n) for each experimental group/condition, given as a discrete number and unit of measurement
☐	☒	A statement on whether measurements were taken from distinct samples or whether the same sample was measured repeatedly
☐	☒	The statistical test(s) used AND whether they are one- or two-sided<br>*Only common tests should be described solely by name; describe more complex techniques in the Methods section.*
☒	☐	A description of all covariates tested
☐	☒	A description of any assumptions or corrections, such as tests of normality and adjustment for multiple comparisons
☐	☒	A full description of the statistical parameters including central tendency (e.g. means) or other basic estimates (e.g. regression coefficient) AND variation (e.g. standard deviation) or associated estimates of uncertainty (e.g. confidence intervals)
☐	☒	For null hypothesis testing, the test statistic (e.g. F, t, r) with confidence intervals, effect sizes, degrees of freedom and P value noted<br>*Give P values as exact values whenever suitable.*
☒	☐	For Bayesian analysis, information on the choice of priors and Markov chain Monte Carlo settings
☐	☒	For hierarchical and complex designs, identification of the appropriate level for tests and full reporting of outcomes
☒	☐	Estimates of effect sizes (e.g. Cohen's d, Pearson's r), indicating how they were calculated

Our web collection on statistics for biologists contains articles on many of the points above.

Software and code

Policy information about availability of computer code

Data collection	The data underlying this article are available at https://www.ncbi.nlm.nih.gov/geo/query/acc.cgi?acc=GSE231993. The IMC raw files, preprocessed files, exported matrix files are deposited into OMIX databases： OMIX001059 （Public available after 2022-03-25 at https://ngdc.cncb.ac.cn/omix/view/OMIX001059）.
Data analysis	Graph Pad prism 8, R 3.6.0., Flow」 o software (TreeStar) 10.6.2, All the codes related to the analysis are publicly available at https://github.com/shaoweinuaa/NC2022_Ulcerative_colitis .

For manuscripts utilizing custom algorithms or software that are central to the research but not yet described in published literature, software must be made available to editors and reviewers. We strongly encourage code deposition in a community repository (e.g. GitHub). See the Nature Portfolio guidelines for submitting code & software for further information.

Data

Policy information about availability of data

All manuscripts must include a data availability statement. This statement should provide the following information, where applicable:

- Accession codes, unique identifiers, or web links for publicly available datasets
- A description of any restrictions on data availability
- For clinical datasets or third party data, please ensure that the statement adheres to our policy

The data underlying this article are available at https://www.ncbi.nlm.nih.gov/geo/query/acc.cgi?acc=GSE231993. The IMC raw files, preprocessed files, exported

matrix files are deposited into OMIX databases： OMIX001059 （Public available after 2022-03-25 at https://ngdc.cncb.ac.cn/omix/view/OMIX001059）.

Human research participants

Policy information about studies involving human research participants and Sex and Gender in Research.

Reporting on sex and gender	We recruited 52 healthy and patients with UC, including 24 females and 28 males with biological sex and self-identified gender
Population characteristics	The age distribution of UC patients and healthy volunteers is 23-70 years old. At the same time, we have made a detailed description of the diagnosis, Mayo Clinic Endoscopic Subscore and the Montreal classification of UC patients (Table S2).
Recruitment	Biopsy specimens from healthy volunteers and UC patients.
Ethics oversight	The study followed the Declaration of Helsinki principles and was approved by the Medical Ethics Committee of the First Affiliated Hospital, Zhejiang University, Hangzhou, China; Tianjin Medical University General Hospital, Tianjin, China; Beijing Chaoyang Hospital, Beijing, China.

Note that full information on the approval of the study protocol must also be provided in the manuscript.

Field-specific reporting

Please select the one below that is the best fit for your research. If you are not sure, read the appropriate sections before making your selection.

☒ Life sciences ☐ Behavioural & social sciences ☐ Ecological, evolutionary & environmental sciences

For a reference copy of the document with all sections, see nature.com/documents/nr-reporting-summary-flat.pdf

Life sciences study design

All studies must disclose on these points even when the disclosure is negative.

Sample size	No calculation was performed. The standard is that samples are enough for experiment conducting.
Data exclusions	No data were excluded.
Replication	All attempts at replication were successful.
Randomization	The experiment groups is just UC and healthy control groups which were diagnosed by experienced pathologist, so there is no need for randomization.
Blinding	The patients were diagnosed with doctors using the diagnostic standard. And the samples just easily categorized to two groups, so there is no need for blinding.

Reporting for specific materials, systems and methods

We require information from authors about some types of materials, experimental systems and methods used in many studies. Here, indicate whether each material, system or method listed is relevant to your study. If you are not sure if a list item applies to your research, read the appropriate section before selecting a response.

Materials & experimental systems

n/a	Involved in the study	
☐	☒	Antibodies
☒	☐	Eukaryotic cell lines
☒	☐	Palaeontology and archaeology
☐	☒	Animals and other organisms
☒	☐	Clinical data
☒	☐	Dual use research of concern

Methods

n/a	Involved in the study	
☒	☐	ChIP-seq
☐	☒	Flow cytometry
☒	☐	MRI-based neuroimaging

Antibodies

Antibodies used	CD45 89 Y D9M8I CST 13917S

IL6 115 In 1A3B4 Proteintech 66146-1-Ig
CD14 141 Pr EPR3653 Abcam ab226121
FoxP3 142 Pr D6O8R CST 12653S
CD16 143 Nd EPR16784 Abcam ab256582
CD69 144 Nd EPR21814 Abcam ab234512
CD4 145Nd EPR6855 Abcam ab181724
CD8a 146 Nd C8/144B Biolegend 372902
Collagen I 147 Sm EPR7785 Abcam ab215969
Cleaved Caspase-3 148 Nd 5A1E CST 9664S
CD31 149 Sm 89C2 CST 3528S
E-cadherin 150 Nd DECMA-1 Biolegend 147302
B7-H4 151 Eu H74 Thermofisher 14-5949-82
VISTA 152 Sm D1L2G CST 64953S
CD7 153 Eu EPR4242 Abcam ab230834
CD169 154 Sm HSn 7D2 Abcam ab18619
CD103 155 Gd EPR4166(2) Abcam ab271889
PD-L1 156 Gd E1L3N CST 13684S
LAG3 158 Gd D2G4O CST 15372S
CD68 159 Tb C8/144B Biolegend 372902
CD11b 160 Gd EPR1344 Abcam ab209970
CD20 161 Dy IGEL/773 Abcam ab213033
CD11c 162 Dy EP1347Y Abcam ab216655
CD15 163 Dy HI98 BD 563872
Granzyme B 164 Dy EPR20129-217 Abcam ab219803
PD-1 165 Ho D4W2J CST 86163S
Ki67 166 Er B56 BD 550609
GATA-3 167 Er D13C9 CST 5852S
HLA-DR 168 Er EDHu-1 Novus NB110-40686
CD45RA 169 Tm HI100 Biolegend 304143
CD3# 170 Er D7A6E CST 85061S
TNF! 171 Yb 7B8A11 Proteintech 60291-1-Ig
IL-1" 172 Yb 2A1B4 Proteintech 66737-1-Ig
CD45RO 173 Yb UCHL1 Biolegend 304239
CD57 174 Yb NK-1 BD 555618
IL-2 Receptor alpha (CD25) 175 Lu EPR6452 Abcam ab215378
Pan-Cytokeratin 176 Yb AE-1/AE-3 Biolegend 914204
Alpha Smooth Muscle Actin 194 Pt 1A4 Biolegend 904601
Vimentin 198 Pt D21H3 CST 5741S
CD45 89 Y D9M8I CST 13917S
IL6 115 In 1A3B4 Proteintech 66146-1-Ig
CD14 141 Pr EPR3653 Abcam ab226121
FoxP3 142 Pr D6O8R CST 12653S
CD16 143 Nd EPR16784 Abcam ab256582
CD69 144 Nd EPR21814 Abcam ab234512
CD4 145Nd EPR6855 Abcam ab181724
CD8a 146 Nd C8/144B Biolegend 372902
Collagen I 147 Sm EPR7785 Abcam ab215969
Cleaved Caspase-3 148 Nd 5A1E CST 9664S
CD31 149 Sm 89C2 CST 3528S
E-cadherin 150 Nd DECMA-1 Biolegend 147302
B7-H4 151 Eu H74 Thermofisher 14-5949-82
VISTA 152 Sm D1L2G CST 64953S
CD7 153 Eu EPR4242 Abcam ab230834
CD169 154 Sm HSn 7D2 Abcam ab18619
CD103 155 Gd EPR4166(2) Abcam ab271889
PD-L1 156 Gd E1L3N CST 13684S
LAG3 158 Gd D2G4O CST 15372S
Pan-cytokeratin AE-1/AE-3 biolegend 914204
CD3e D7A6E CST 85061S
CD11b EPR1344 abcam ab209970
CD20 H1 BD 555677
CD11c EP1347Y abcam ab216655
CD45RO UCHL1 biolegend 304239
CD45RA HI100 biolegend 304143
CD4 EPR6855 abcam ab181724
CD8 C8/144B biolegend 372902
CD45 D9M8I CST 13917BF
TNF-! 7B8A11 Proteintech 60291-1-Ig

CD45 89Y HI30 Biolegend 304045
CD3 115In UCHT1 Biolegend 300443
Ki-67 139La SolA15 Biolegend 350523
CD56 141Pr HCD56 Biolegend 318345
TCR gd 142Nd 5A6.E9 Thermofisher TCR1061
CCR6 143Gd G034E3 Biolegend 353427
CD134 144Nd BER-ACT35 Biolegend 350015
IgD 145Nd IA6-2 Biolegend 348235
CD7 146Nd CD7-6B7 Biolegend 343111
Tim-3 147Sm F38-2E2 Biolegend 345019
CD19 148Nd HIB19 Biolegend 302247
CD25 149Sm M-A251 Biolegend 356102
CD223 150Nd 874501 R&D MAB23193
CD20 151Eu 2H7 Biolegend 302343
CD103 152Sm B-Ly7 Thermofisher 14-1038-82
CD161 153Eu HP-3G10 Biolegend 339919
TIGIT 154Sm A15153G Biolegend 372702
CD45RA 155Gd HI100 Biolegend 304143
CCR4 156Gd L291H4 Biolegend 359402
CD27 (metal-labled) 157Nd O323 PLTTECH 02.01.H.0000037
CCR7 158Gd G043H7 Biolegend 353237
GITR 159Tb 621 Biolegend 311602
CD28 160Gd CD28.2 Biolegend 302937
CTLA4 161Dy 14D3 Thermofisher 14-1529-82
Foxp3 162Dy PCH101 Thermofisher 14-4776-82
CD57 164Dy HNK-1 Biolegend 359602
Tbet 165Ho 4B10 Biolegend 644825
CXCR3 166Er G025H7 Biolegend 353733
ICOS 167Er C398.4A Biolegend 313502
4-1BB 168Er 4B4-1 Biolegend 309802
CD45RO 169Tm UCHL1 Biolegend 304239
CD127 170Er A019D5 Biolegend 351337
CD69 171Yb FN50 Biolegend 310939
CD138 172Yb DL-101 Biolegend 352311
GranzymeB 173Yb GB11 Thermofisher MA1-80734
PD-1 174Yb EH12.2H7 Biolegend 329941
BTLA 175Lu MIH26 Biolegend 344502
HLA-DR 176Yb L243 Biolegend 307651
CD4 (metal-labled) 197Au RPA-T4 PLTTECH 02.01.H.0000643
CD8 198Pt RPA-T8 Biolegend 301053
CD45 HI30 BD 563716
CD11b 1CRF44 BD 562721
CD4 RPA-T4 BD 557871
CD8 RPA-T8 BD 557746
CD19 SJ25C1 BD 563549
CD3 SK7 Biolegend 981002
CD68 Y1/82A Biolegend 333808
HLA-DR L243 Biolegend 307626
EpCAM 9C4 Biolegend 324208
CCR2 K036C2 Biolegend 357204
CD45 30-F11 BD 563053
CD11bM1/70 BD 562287
MHCII (I-A/I-E) M5/114.15.2 BD 562564
CD4 GK1.5 BD 552051
F4/80 BM8 Biolegend 123114
CD3# 17A2 Biolegend 100203
Ly6C HK1.4 Biolegend 128036
Ly6G 1A8 Biolegend 127605
CD8a 53-6.7 Biolegend 100722
TNF! MP6-XT22 Thermofisher 12-7321-82
CD68 159 Tb C8/144B Biolegend 372902
CD11b 160 Gd EPR1344 Abcam ab209970
CD20 161 Dy IGEL/773 Abcam ab213033
CD11c 162 Dy EP1347Y Abcam ab216655
CD15 163 Dy HI98 BD 563872
Granzyme B 164 Dy EPR20129-217 Abcam ab219803
PD-1 165 Ho D4W2J CST 86163S
Ki67 166 Er B56 BD 550609

GATA-3 167 Er D13C9 CST 5852S
HLA-DR 168 Er EDHu-1 Novus NB110-40686
CD45RA 169 Tm HI100 Biolegend 304143
CD3# 170 Er D7A6E CST 85061S
TNF! 171 Yb 7B8A11 Proteintech 60291-1-Ig
IL-1" 172 Yb 2A1B4 Proteintech 66737-1-Ig
CD45RO 173 Yb UCHL1 Biolegend 304239
CD57 174 Yb NK-1 BD 555618
IL-2 Receptor alpha (CD25) 175 Lu EPR6452 Abcam ab215378
Pan-Cytokeratin 176 Yb AE-1/AE-3 Biolegend 914204
Alpha Smooth Muscle Actin 194 Pt 1A4 Biolegend 904601
Vimentin 198 Pt D21H3 CST 5741S

Validation

IHC validation and validation statements on manufacture's website

Animals and other research organisms

Policy information about studies involving animals; ARRIVE guidelines recommended for reporting animal research, and Sex and Gender in Research

Laboratory animals

C57BL/6J mice were purchased from the Model Animal Research Center of Nanjing University (China). CCR2 Knockout mice were bred in-house. Only male mice were used for the mouse colitis experiment. All mice were bred in an SPF facility under the regulation of the Institutional Animal Care & Use Committee (IACUC).

Wild animals

No wild animals were used.

Reporting on sex

Only male mice were used for the mouse colitis experiment.

Field-collected samples

All mice were bred in an SPF facility under the regulation of the Institutional Animal Care & Use Committee (IACUC).

Ethics oversight

Animal experiments were approved by the Animal Care and Medical Ethics Committee of the First Affiliated Hospital, Zhejiang University.

Note that full information on the approval of the study protocol must also be provided in the manuscript.

Flow Cytometry

Plots

Confirm that:

- [x] The axis labels state the marker and fluorochrome used (e.g. CD4-FITC).
- [x] The axis scales are clearly visible. Include numbers along axes only for bottom left plot of group (a 'group' is an analysis of identical markers).
- [x] All plots are contour plots with outliers or pseudocolor plots.
- [x] A numerical value for number of cells or percentage (with statistics) is provided.

Methodology

Sample preparation

Tissue obtained by biopsies or mice colon were digested in a culture medium supplemented with 0.6 mg/mL collagenase IV (17104019, Gibco) and 0.01 mg/mL DNase I (11284932001, Merck) in a 170-rpm constant temperature shaker (Eppendorf) at 37°C for 1 hour. Digested tissues were passed through a 70 μm cell strainer (352340, BD) to acquire single-cell suspensions before centrifuging at 300 g for 5 min and resuspending in 35% Percoll (P4937, Sigma). After another centrifugation at 500 g for 5 min, cells were resuspended in 10 mL of blood lysis buffer (555899, BD) for 10 min at RT to eliminate red blood cells. Cell suspensions were centrifuged at 300 g for 5 min and then incubated with 2.5 μg/mL Fc blocker (156604 for mouse; 422302 for human, BioLegend) on ice for 15 min. The suspensions were further incubated with fluorochrome- or metal-labeled antibodies at 4°C for 30 min. The antibody panel is listed in Supplementary Table S1. The samples were washed and resuspended in PBS and supplemented with 2% FBS. For intracellular staining, BD kits (555028) were used. For FACS analysis, a five-laser flow cytometer (BD Bioscience, Fortessa) was applied. The FACS data were analyzed with FlowJo software (TreeStar).

Instrument

A five-laser flow cytometer (BD Bioscience, Fortessa)

Software

The FACS data were analyzed with FlowJo software 10.6.0(TreeStar).

Cell population abundance

The samples were washed and resuspended in PBS as the concentration of 1×10^6 / ml and supplemented with 2% FBS.

Gating strategy

All gating strategy were elaborated in figures and the text.

- [x] Tick this box to confirm that a figure exemplifying the gating strategy is provided in the Supplementary Information.
